# An overview of the trypanosomatid (Kinetoplastida: Trypanosomatidae) parasites infecting several mammal species in Colombia

**DOI:** 10.1186/s13071-022-05595-y

**Published:** 2022-12-16

**Authors:** Adriana C. Castillo-Castañeda, Luz H. Patiño, Maria Fernanda Zuñiga, Omar Cantillo-Barraza, Martha S. Ayala, Maryi Segura, Jessica Bautista, Plutarco Urbano, Jeiczon Jaimes-Dueñez, Juan David Ramírez

**Affiliations:** 1 Centro de Investigaciones en Microbiología y Biotecnología-UR (CIMBIUR), Facultad de Ciencias Naturales, Universidad del Rosario, Bogotá, Colombia; 2 Grupo de Biología y Control de Enfermedades Infecciosas (BCEI), Universidad de Antioquia, Medellín, Colombia; 3 Grupo de Parasitología, Instituto Nacional de Salud, Bogotá, Colombia; 4 Grupo de Investigaciones Biológicas de La Orinoquía, Universidad Internacional del Trópico Americano (Unitropico), Yopal, Colombia; 5 Grupo de Investigación en Ciencias Animales GRICA, Facultad de Medicina Veterinaria y Zootecnia, Universidad Cooperativa de Colombia UCC, Bucaramanga, Colombia; 6 Department of Pathology, Molecular and Cell-Based Medicine, Molecular Microbiology Laboratory, Icahn School of Medicine at Mount Sinai, New York, NY USA

**Keywords:** Amplicon-based NGS, Sanger, Mammals, Trypanosomatids, Coinfection, Diversity

## Abstract

**Background:**

Trypanosomatids are among the most critical parasites for public health due to their impact on human, animal, and plant health. Diseases associated with these pathogens manifest mainly in poor and vulnerable populations, where social, environmental, and biological factors modulate the case incidence and geographical distribution.

**Methods:**

We used Sanger and amplicon-based next-generation sequencing (NGS) in samples from different mammals to identify trypanosomatid infections in several departments in Colombia. A total of 174 DNA samples (18 humans, 83 dogs, and 73 wild mammals) were analyzed by conventional PCR using a fragment of the heat shock protein 70 (*Hsp70*) gene and Sanger sequenced the positive samples. Twenty-seven samples were sent for amplicon-based NGS using the same gene fragment. Data obtained were used to perform diversity analyses.

**Results:**

One hundred and thirteen samples were positive for PCR by *Hsp70* fragment; these corresponded to 22.1% *Leishmania* spp., 18.6% *L. amazonensis*, 9.7% *L. braziliensis*, 14.2% *L. infantum*, 8% *L. panamensis*, and 27.4% *Trypanosoma cruzi*. Comparison of the identified species by the two sequencing technologies used resulted in 97% concordance. Alpha and beta diversity indices were significant, mainly for dogs; there was an interesting index of coinfection events in the analyzed samples: different *Leishmania* species and the simultaneous presence of *T. cruzi* and even *T. rangeli* in one of the samples analyzed. Moreover, a low presence of *L. braziliensis* was observed in samples from wild mammals. Interestingly, to our knowledge, this is the first report of *Leishmania* detection in *Hydrochaeris hydrochaeris* (capybara) in Colombia.

**Conclusions:**

The *Hsp70* fragment used in this study is an optimal molecular marker for trypanosomatid identification in many hosts and allows the identification of different species in the same sample when amplicon-based sequencing is used. However, the use of this fragment for molecular diagnosis through conventional PCR should be carefully interpreted because of this same capacity to identify several parasites. This point is of pivotal importance in highly endemic countries across South America because of the co-circulation of different genera from the Trypanosomatidae family. The findings show an interesting starting point for One Health approaches in which coevolution and vector-host interactions can be studied.

**Graphical Abstract:**

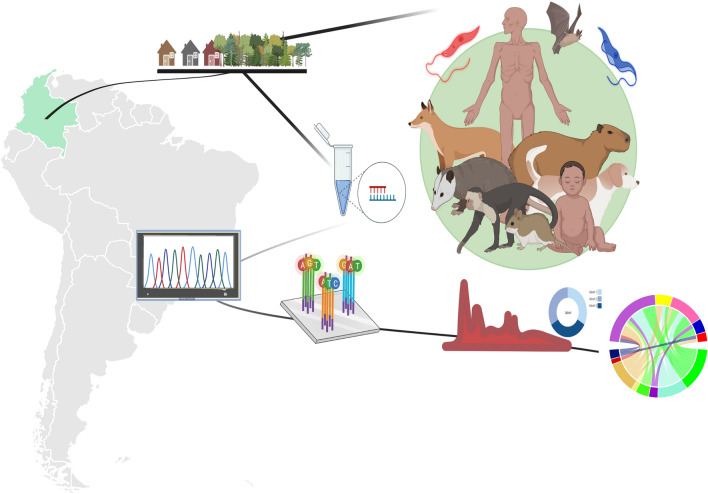

**Supplementary Information:**

The online version contains supplementary material available at 10.1186/s13071-022-05595-y.

## Background

Kinetoplastid parasites have been a primary worldwide concern for centuries, where *Leishmania* and *Trypanosoma* stand out as the most critical genera [1]. These have tremendous importance for public health because of their impact on human and animal diseases, reflected in economic losses associated with morbidity, mortality, cost overrun in health systems, and investment in prevention programs, among others [2]. Furthermore, for plants, *Phytomonas* spp. is associated with damage to coffee, oil palm, and coconut plantations, with economic effects due to crop failures, pesticides use, loss of cultivable fields, and biodiversity, leading to ecological imbalance [3–5].

Human and animal diseases associated with these pathogens have great significance for the World Health Organization (WHO), considering that they are included in the 2030 agenda for the elimination of neglected tropical diseases (NTDs) [6]. For both leishmaniasis and trypanosomiasis, poverty, vulnerability [7], environmental [8], social [9–13], and biological factors [1] modulate the geographic distribution of the pathogens, their vectors, and consequently the incidence of human cases. In mammals, trypanosomatids are transmitted mainly by vectors; however, oral infections represent a vital infection route in the wild transmission cycle. For *Leishmania* spp., transmission is by the bite of infected female phlebotomine sand flies [14], having three clinical manifestations in humans: cutaneous, mucocutaneous, and visceral leishmaniasis (VL) [15]. In the case of *Trypanosoma* spp., the vectorial transmission is mediated by triatomines for *T. cruzi* and *T. rangeli* and tsetse flies for *T. brucei*, causing asymptomatic infections or acute disease that can evolve to a chronic phase in humans [1]. The severity of these parasitic diseases has been related to the infecting species, infection route, patient's immunological response, comorbidities, and treatment opportunities [16, 17].

Sanger sequencing has helped the study of *Leishmania* spp., *Trypanosoma* spp., their vectors, and their feeding preferences [18–24]. Indeed, Asia and the Mediterranean basin have reported the presence of *Trypanosoma* spp. DNA in phlebotomines [25–27]. Also, Sanger technology has helped determine the causal agents of leishmaniasis and trypanosomiasis in urban and periurban transmission cycles [24, 28–32]. Likewise, the DNA of trypanosomatids has also been identified in several mammals of the sylvatic cycle, such as rodents [33–35], didelphids [36, 37], marsupials [38], bats [39–43], and primates [44, 45]. Such analyses in vectors and reservoirs are highly relevant for public health, considering they allow determining the incidence of parasitic species in the transmission hotspots and their geographical distribution as well as the study of the genetic diversity of *Leishmania* spp. [46–49] and *Trypanosoma* spp. [50–52] worldwide.

Easy access to next-generation sequencing (NGS) technologies and methodologies, such as amplicon-based NGS, has allowed generating and analyzing large and complete amounts of data on the parasites [53–57]. For leishmaniasis expressly, this methodology has provided numerous highlights, for instance, the identification of *Leishmania* species in new geographic regions [58], infection indices and feeding preferences in vectors [25, 59], and identification of the most influential reservoirs in the transmission cycles [30, 40, 60, 61]. Regarding trypanosomiasis, NGS has facilitated the study of *T. cruzi* and *T. rangeli* genetic diversity [56], lineage associations in asymptomatic, acute, and chronic cases of Chagas disease [62], and identification of multiple feeding preferences in triatomines [53], among others, and detected coinfection events by different parasitic species in a single host [58, 63].

Although leishmaniasis and Chagas disease are important because of their incidence and wide geographical distribution [18, 64], there are few investigations related to the study of these agents in mammals, especially in different transmission cycles in Colombian departments, with an active circulation of the parasites. Therefore, using NGS (amplicon-based) and Sanger, we aimed to study and improve the understanding of the transmission cycles of trypanosomatids in samples obtained from different wild and domestic mammals in many departments in Colombia. This study has the additional purpose of encouraging the use of this type of research on different players in the life cycle of parasites in endemic countries, hence generating updated data useful for government stakeholders for the promotion and prevention of these diseases using a One Health context.

## Methods

### Samples

**Fig. 1:**
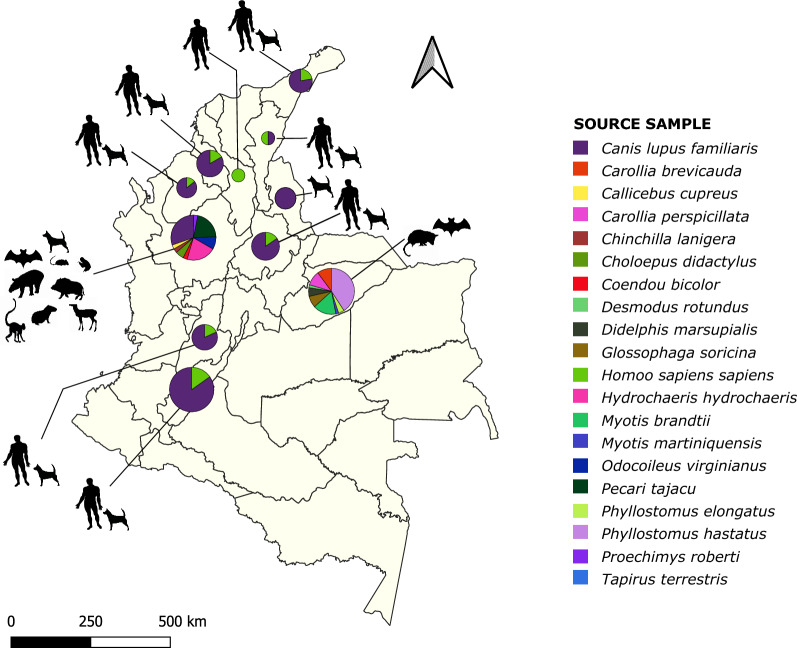
Geographical and biological distribution of the different samples analyzed in this study. Plot size is proportional to the total number of samples per department, and each mammal species is represented by a single color

A total of 174 samples were included by convenience in this study: 18 from humans with VL diagnosis from the departments of Bolívar, Córdoba, Huila, La Guajira, Norte de Santander, Santander, Sucre, and Tolima; 83 of domestic dogs (*Canis lupus familiaris*) from Antioquia, Santander, La Guajira, Cesar, Córdoba, Huila, Norte de Santander, Santander, Sucre, and Tolima; 73 of wild mammals (*Callicebus cupreus*, *Carollia brevicauda*, *Carollia perspicillata*, *Chinchilla lanigera*, *Choloepus didactylus*, *Coendou bicolor*, *Desmodus rotundus*, *Didelphis marsupialis*, *Glossophaga soricina*, *Hydrochaeris hydrochaeris*, *Myotis brandtii*, *Myotis martiniquensis*, *Odocoileus virginianus*, *Pecari tajacu*, *Phyllostomus elongatus*, *Phyllostomus hastatus*, *Proechimys roberti*, and *Tapirus terrestris*) from Antioquia and Casanare (Fig. 1; Additional file 1: Table S1). Most departments are endemic for leishmaniasis and Chagas disease. The geographic distribution by department in Colombia is shown in the supplementary information (Additional file 2: Fig. S1).

Human samples were obtained from two sources: serum via venipuncture and bone marrow aspirate smear slides. From canines, these were obtained by anticoagulated total blood with EDTA (venipuncture) or serum in a dry tube. The samples were anticoagulated whole blood with EDTA or collected in FTA cards for wild mammals. All animals were captured with the minimum damage possible. The wild mammals were anesthetized with 20 mg/kg body weight ketamine (Ketalar, Parke Davis, Morris Plains, NJ, USA), and blood was obtained via venipuncture. For bats, only 300 µl whole blood was collected. All the plasma and serum samples were conserved at –80 °C until their processing; FTA cards and slides were stored at environmental temperature and humidity for optimal storage conditions.

### DNA extraction

All the biological samples collected were processed using the High Pure PCR Template Preparation Kit (Roche Life Science, Mannheim, Germany) following the protocol described by the manufacturer. The slide samples were submerged in xylol to clean the immersion oil traces; next, 200 µl lysis buffer was added for 10 min, and the smear was carefully removed and put into a microtube to start the DNA extraction. DNA concentration was determined using NanoDrop ND-1000 spectrophotometer (Thermo Fisher Scientific Inc., Waltham, MA, USA), and the DNA quality and integrity were checked through gel electrophoresis in agarose 1%. Samples were conserved at -80 °C until processing.

### Molecular test and *Trypanosoma* species identification by Sanger sequencing

As previously reported, a 337-bp region of the *Hsp70* gene for both *Trypanosoma* and *Leishmania* was amplified by conventional PCR [65, 66]. Amplicon products were analyzed by gel electrophoresis in 2% agarose. Those products with gel band presence (positive for *Hsp70*) were purified with EXOSAP (Affymetrix, Santa Clara, CA, USA) and sent for sequencing by the dideoxy-terminal method in an automated capillary sequencer (AB3730; Applied Biosystem, Foster City, CA, USA). The sequences were submitted to BLASTn using the NCBI platform [65]. Subsequently, the DNA of all the samples identified with some species of *Leishmania* that met the quality requirements for Novogene were sent for amplicon-based sequencing by Illumina. Additionally, 60% of the samples were BLAST-identified as *T. cruzi*, and ten samples from canines with visceral leishmaniasis diagnosis were also sent for sequencing. In the first scenario to assess the co-infection, when Sanger sequencing identified *T. cruzi* as the main infecting parasite, the second validated the possibility that amplicon-based NGS had more power to detect target reads.

### Amplicon-based next-generation sequencing

Genomic DNA (> 200 ng/μl) from humans, canines, and wild mammals was sent to amplicon-based sequencing by Illumina (Novogene, Beijing, China). The primers used were the same for the conventional PCR, forward (5'AGGTGAAGGCGACGAACG) and reverse (5'CGCTTGTCCATCTTTGCGTC), following the protocol, as reported before [58].

### Bioinformatics analysis

The FASTA files from the *Hsp70* raw sequences were filtered using QIIME software [67], considering the parameters described before [53]. Then, barcode trimming and forward and reverse sequence merging were made. Then, another quality filter was made for the merged files. The reads that passed the quality filters were compared against an in-house database, which contains sequences for the *Hsp70* 337-bp fragment of kinetoplastids available in GenBank [58]. The database includes species of *Leishmania*, *Trypanosoma*, and *Leptomonas*. The local BLASTn was made with a threshold of 95% identity and an e-value of 10. Of those species that matched, only the ones with abundance of the total reads per sample of > 3% significance were considered. Quantitative results were plotted using R software version 3.6.2 and the Sankey diagram package available at www.online.visu-alparadigm.com.

### Statistical analysis

The qualitative variables were clustered by frequency and proportions according to the parasite species and coinfection patterns depending on the data obtained from amplicon-based sequencing. Considering the normality of the data, a Chi-square test (χ²) was made to analyze the relation between mammal-parasite and origin (department)-parasites. The statistical analysis was executed in R software (RStudio Team 2019). Two-sided significance tests and *P*-value < 0.05 were established. Moreover, to analyze the correspondence among the parasites reported by *hsp70* sequencing by Sanger and the amplicon-based sequencing, a kappa (κ) coefficient was calculated using STATA11 with 0.05 significance.

## Results

### Trypanosomatid identification by Sanger sequencing

**Fig. 2:**
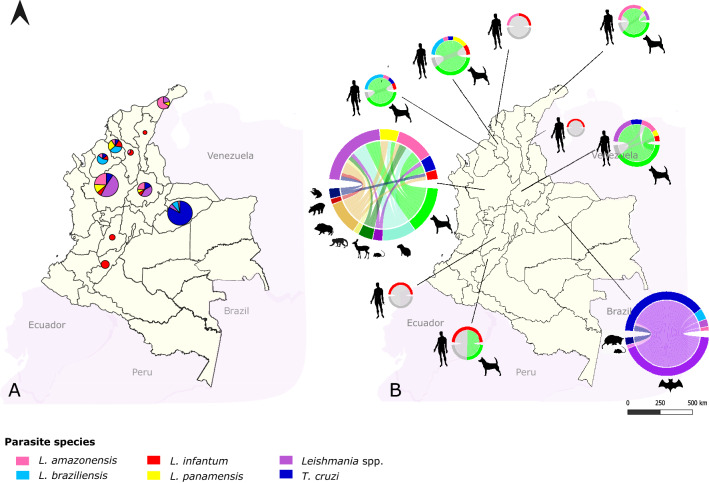
Results of the biological and geographical distribution of trypanosomatid species identified by Sanger sequencing. **A** Parasite species found for each animal analyzed per department (**B**). Plot size is proportional to the total number of samples analyzed per department. For each plot from panel (**B**), the size of the inner joining line among mammals (lower half) and parasite species (upper half) is proportional to sample number. Each parasite species is represented by a single color

Overall, 64.9% of the total samples used in the study (113/174) had amplification for *Hsp70* by conventional PCR (Additional file 1: Table S1), of which 12.2% (14/18) were from humans, 40.4% (46/83) from canines, and 47.4% (54/73) from wild mammals. Results obtained from BLASTn (Fig. 2; Additional file 3: Table S2) show that Colombia has a wide variety of *Leishmania* species, mainly in the departments with co-circulation of *T. cruzi*. For mammal species, *L. infantum* (71.4%) and *L. amazonensis* (21.4%) were the most frequent species in human samples with VL diagnosis; for canines, they were *L. amazonensis* (26.1%), *L. braziliensis* (17.4%), and *Leishmania* spp. (21.8%). For wild mammals, they were *T. cruzi* (47.2%) and *Leishmania* spp. (26.4%), *L. amazonensis* (11.3%), and *L. braziliensis* (5.7%) (Fig. 2). Furthermore, considering the origin of the samples, a high diversity of parasitic species was found for each animal, *T. cruzi* and *Leishmania* spp. being the most prevalent, with 27.4% and 22.1%, respectively (Fig. 2). The former was more frequent in Casanare, where the samples were collected mostly from bats.

### *Hsp70* sequencing by amplicon-based NGS analysis

**Fig. 3:**
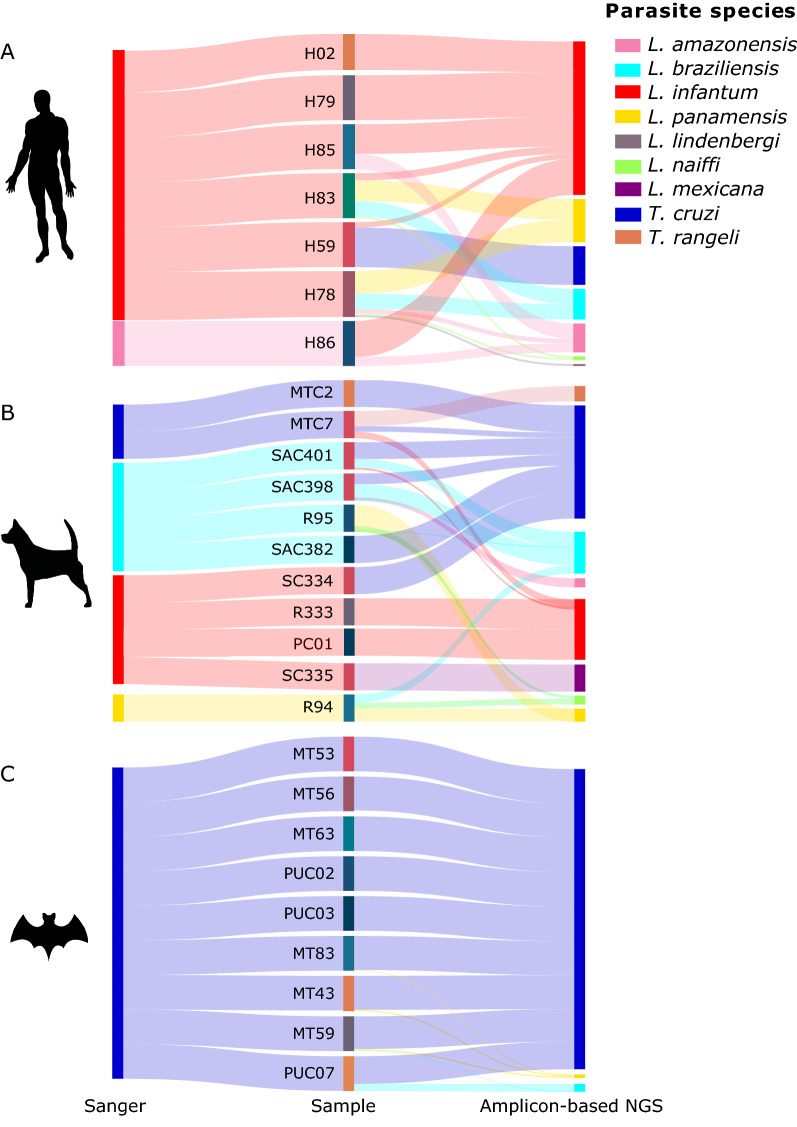
*Leishmania* and *Trypanosoma* species correspondence reported by Sanger sequencing and reads obtained by amplicon-based NGS from human samples (**A**), dogs (**B**), and wild mammals (**C**). The size of the inner joining line among samples and parasites is proportional to the reads percentage found in the analyzed sample. Each parasite species is represented by a single color

Only 118 samples met the requirement of the DNA concentration (≥ 200 ng/μl) for Illumina, of which 22.9% (27/118) were optimal for analysis. Subsequent sequencing of the 337-bp *Hsp70* fragment by Illumina generated between 134,316 and 179,347 paired-end reads. The bioinformatic analysis revealed that for 96.2% of the samples (26/27), > 85% of the reads had a minimum coverage > q20 (initial quality filter). The exception was a canine from Sucre (R95), in which only 41% of the reads successfully passed the initial quality control. Furthermore, the taxonomic assignment made with local BLASTn was performed with high-quality reads per sample using > 38% of the reads generated at the beginning for almost all cases. Unexpectedly, the taxonomic assignation for animal samples C334, C335, PUC07, and SAC382 (Fig. 3) resulted in individual matches for 35, 108, 608, and 234 reads with the species included in the database used.

### Concordance between Sanger and amplicon-based NGS results

It is known that the two sequencing methods used in this study have different methodological principles, scope, and output. However, we compared whether the species (unique) obtained with Sanger were included or not in the unique or multiple species obtained with amplicon-based NGS. We showed a general concordance between the two sequencing techniques of 97% and a kappa coefficient of 0.8–1.0 by comparing the identified species.

### In amplicon-based NGS analysis, coinfection events in VL patients' samples and canines were frequent

The coinfection events were more frequent in human and canine samples compared to samples from wild mammals. Coinfection was identified from the human samples (5/7); infection frequency by *L. infantum* was 85.8%, *L. amazonensis* 42.6%, *L. braziliensis, L. panamensis*, and *L. naiffi* 28.6%, with 14.3% for *L. lindenbergi* along with *T. cruzi*. Double infection events were detected: *T. cruzi/L. infantum* (1 sample) and *L. amazonensis/L. infantum* (2 samples) and multiple infection by *L. infantum/L. braziliensis/L. panamensis/L. naiffi* and *L. amazonensis/L. braziliensis/L. panamensis/L.naiffi/L. lindenbergi* in the same patient (Fig. 3A); a single infection by *L. infantum* in humans was present in 28.6% (2/7) of the samples, in concordance with Sanger reports**.** Canine samples presented a wide diversity of *Leishmania* species, with a single infection in around 50%, *T. cruzi* infection in three samples, *L. infantum* and *L. mexicana* in one sample each, and triple infection by *T. cruzi/T. rangeli/L. infantum* in a canine from Santander; the samples from Sucre showed multiple infections: *L. braziliensis/L. panamensis/L. naiffi* and two canines by *T. cruzi/L. amazonensis/L. braziliensis* and *T. cruzi/L. infantum/L. braziliensis*, respectively (Fig. 3B).

### *Trypanosoma cruzi* prevails in wild mammals

One hundred percent of wild mammals had reads for *T. cruzi* in the amplicon-based NGS. Double infection by *T. cruzi* with *L. panamensis* or *L. braziliensis* was present in three samples and triple infection by *T. cruzi* along with *L. braziliensis/L. panamensis* in one sample. Single infection by *T. cruzi* was present in 44.4% of the samples (Fig. 3C).

### Statistical analysis

The statistical analysis did not reveal statistically significant differences between the coinfection and single-infection groups analyzed (Mann-Whitney-Wilcoxon test, *P* = 0.07, 0.87, and 0.566) or among species (Kruskal-Wallis test, *P* = 0.31, 0.195 and 0.567).

Chi-squared tests and Fisher exact tests were performed to evaluate a potential association between the categorical variables and for the relation between department and species (*P* = 0.038) and chi-squared test (*P* = 0.022) for parasite species vs. mammal.

### Diversity analysis

Analyzing the alpha diversity of the samples by amplicon-based NGS, statistically significant differences among the three groups were analyzed (Shannon index: *P* < 0.0001; Simpson index *P* = 0.0001). Humans and dogs presented the most diversity (Shannon index 1.14, 1.17 for humans and 1.04 for canines) and dominance (Simpson index 0.64 and 0.62, respectively) compared with the wild mammals where the obtained values were close to zero (Additional file 4: Table S3). This comparison revealed statistically significant differences in alpha diversity between humans and wild mammals and dogs and wild mammals.

## Discussion

The diversity of *Leishmania* species found using *Hsp70* amplicons by Sanger sequencing agree with those expected for patients with VL diagnosis (Additional file 3: Table S2), which historically have been *L. infantum* for the Americas [68–70]. We also found *L. amazonensis* in patient samples from La Guajira, Santander, and Bolívar (Fig. 2), an atypical event that has been previously reported in humans [71] and dogs [72, 73]. For humans, *L. major* and *L. tropica* have been the principal non-*L. donovani* complex species reported in the Old World, while in the Americas they have been *L. braziliensis, L. mexicana*, and *L. amazonensis*. In both geographical contexts, HIV is the main factor described for coinfection events in immunocompromised patients [74]. In La Guajira, the samples collected came from a new hotspot of VL in Hatonuevo municipality, in which *L. amazonensis* was detected in both humans and canines (Additional file 3: Table S2). This allowed us to consider the potential existence of a new VL hotspot solely associated with *L. amazonensis*, even though further investigations must include more comprehensive sampling, vectors, and parasite isolation to prove *L. amazonensis*'s capacity for visceral tropism.

We also found a high diversity of *Leishmania* species in dogs and wild mammals, besides the presence of *T. cruzi* in animals from the different departments of Colombia (Fig. 2). The latter has high prevalence in regions highly endemic for Chagas disease [75, 76] such as Casanare. These findings showed that, regarding the NTD elimination programs focused on vector control and diagnosis/treatment in humans, the pathogen transmission remains enzootic [77]. The above is alarming considering the increasing sylvatic niche fragmentation, which also increases the risk of outbreaks, sylvatic parasite species circulation in urban transmission cycles, and the adaptation of the pathogens according to the availability of vectors and hosts [78, 79]. This problem has been acknowledged and studied in endemic regions in the Brazilian Amazon, keeping in mind their local context and associated variables to strengthen One Health intervention programs [80].

Furthermore, the presence of different *Leishmania* species is related to Colombia's high biodiversity [81], where the vectors' diversity and ecological niches [44, 82] allow the maintenance of *L. panamensis*, *L. amazonensis*, and *L. braziliensis* in sylvatic, urban, and periurban transmission cycles. Additionally, differently than expected for *L. braziliensis* and *L. panamensis* (the most prevalent species of cutaneous leishmaniasis in active military populations) [58, 83], we found a low number of wild mammals infected with these species in Antioquia. On the other hand, *L. amazonensis* predominates, and *L. infantum* was identified in *Pecari tajacu* (collared peccary) and *Choloepus didactylus* (Linnaeus's two-toed sloth). In all *Hydrochaeris hydrochaeris* (Capybara) samples, *L. amazonensis, L. panamensis*, and *Leishmania* spp. were identified, as previously reported in other countries [84, 85]. This represents the first report in Colombia highlighting the need to conduct studies on this species, which represents an exotic source of consumable meat in the region.

For Casanare, *T. cruzi* was the main trypanosomatid detected. *Leishmania* was identified in four animals (3 *L. braziliensis* and 1 *L. amazonensis*) (Fig. 2A). These findings suggest that the vector species distribution could determine the patterns according to specific environmental conditions, their feeding preferences, and the availability of specific reservoirs [86–89]. Therefore, by analyzing the data for Antioquia (Fig. 2; Additional file 3: Table S2), the possibility can be suggested that the lack of identification of *L. braziliensis* was determined by the mammal species sampled in this study, while for Casanare, the hypothesis could explain how, despite finding bats infected with *L. braziliensis* and *L. amazonensis* (Figs. 2, 3C) and the presence of the circulation of *Lutzomyia gomezi* in the department [90], no autochthonous cases of leishmaniasis have been reported according to official data from the Colombian Disease Surveillance System (SIVIGILA). It can be assumed that the phlebotomines play an essential role in disease modeling in humans and the maintenance of the parasite’s enzootic cycle, as has been demonstrated in endemic regions for cutaneous and visceral leishmaniasis in Brazil, Spain, and Iran [91–93]. However, a broader sampling and inclusion of more mammals, as well as the parallel study of phlebotomine circulation, distribution, and feeding preferences, are needed.

Additionally, two human and canine samples from Sucre presented the highest diversity index (Additional file 4: Table S3), where we identified coinfection events with *L. naiffi* and *L. lindenbergi* (Fig. 3A, B), as recently reported [58, 63]. The high species richness of *Leishmania* in a single individual could be associated with the proximity of dwellings to forests, with a circulation of different vector species such as *Lu. longipalpis, Lu. evansi*, and *Lu. gomezi* [90, 94], human and canine mobilization to the forests to search for natural resources, and military and illegal groups in this country zone [9, 63, 65, 83]. All these factors make the vector-human-reservoir-pathogen interaction more accessible, maintaining the zoonotic and enzootic transmission cycles of *Leishmania* spp. Some authors have concluded, for instance, that the circulating phlebotomine sand fly species are critical for the vectorial transmission of *Leishmania* spp. [95]; likewise, the mammals' role in parasite transmission concerns the vector, their meal preferences, and feeding behavior [96, 97].

Considering the identified parasite species versus those expected in wild mammals and coinfection events, a new scenario is opening showing the need for research on the following topics: (i) the role of domestic/wild mammals and vectors in the maintenance of transmission cycles, which has been studied and proposed in mathematical models for different vector-borne diseases [98–100]; (ii) the domiciliation transition of vectors in specific areas, phenomena highly relevant for American trypanosomiasis and VL in recent years [101–103]; (iii) the possibility of the genetic recombination of the different actors implicated in the parasites’ life cycle, not just for the vector context [104–106]. The latter must transcend the world view of human diseases and recognize their importance and the veterinary diseases that must be equally prioritized in the public health systems [107]. Therefore, considering our results, we highlight the relevance and usefulness of transmission scenarios in Casanare, Antioquia, and Sucre to understand these phenomena's ecological dynamics better.

On the other hand, we found coinfection by *L. infantum, T. rangeli*, and *T. cruzi* in a canine in Santander (Fig. 3B), a department with a high incidence of Chagas disease. It is known that *T. cruzi* and *T. rangeli* share mammal hosts, and their geographical distribution overlaps with the finding of infected mammals and triatomines [108, 109]. This triple coinfection was previously reported in *Tamandua tetradactyla*, a wild mammal [110]. In humans, even though *T. cruzi-T. rangeli* coinfection affects Chagas disease diagnosis, the cases are underestimated as *T. rangeli* has been detected in primates, bats, rodents, marsupials, and dogs in Brazil, Colombia, and Venezuela [110, 113–118].

It is relevant to discuss the benefits and limitations of amplicon-based sequencing and the specificity of the *Hsp70* gene fragment used. In the first place, NGS technologies and the inclusion of new methodologies, such as amplicon-based ones, offer benefits in cost reduction and obtain quick and sensitive high-throughput data [117, 118], allowing the use of different target genes simultaneously [119, 120]. The time between sample processing and data collection is less than for conventional PCR and Sanger sequencing [121]. One of the critical points in sequencing success is the pre-analytics phase; therefore, the samples used in this study (serum, total blood, and bone marrow aspirate smear) determined the DNA integrity and concentration, which directly influence the success of NGS sequencing [122, 123]. Moreover, the biological influence of parasitic load in mammals and the copy number of the *Hsp70* gene should be considered. This explains the sample percentage that could be evaluated by amplicon-based sequencing (Fig. 3; Additional file 3: Table S2). Second, the *Hsp70* gene allows the identification of *Leishmania* and *Trypanosoma* [124], which is an advantage for studying samples from endemic regions for both parasites.

Nevertheless, the *Hsp70* gene sensitivity is not optimal for use as a diagnostic marker. One of our limitations was not being able to include more sensitive markers, such as satellite DNA for *T. cruzi* [125] or 18S for *Leishmania* [126], to determine whether those 60 samples were indeed negative. However, we want to emphasize that the main objective was to depict the infective species, so we chose the *Hsp70* marker. Finally, considering the rising availability of data from outstanding databases such as NCBI, searching for a more sensitive genetic marker to discriminate among these trypanosomatid species through Illumina sequencing or even Oxford Nanopore should be prioritized.

## Conclusions

The present study describes the infection by trypanosomatids in samples from humans, dogs, and wild mammals, using Sanger and amplicon-based sequencing of a coding fragment of the *Hsp70* gene. We confirmed the vast diversity of *Leishmania* species found in the different samples obtained in many departments of Colombia, the presence of *T. cruzi* in bats and dogs, and the occurrence of coinfections.

## Supplementary Information

**Additional file 1:** **Fig. S1.** Map showing the different departments and the capital district of Colombia.

**Additional file 2: Table S1.** Information on collected samples per department, sample code, and mammal species.

**Additional file 3:** **Table S2.** BLASTn results for the 337bp *Hsp70* gene fragment of the samples analyzed herein.

**Additional file 4:** **Table S3.** Shannon and Simpson index values from the different species found in the analyzed samples by amplicon-based NGS.

## Data Availability

All data generated or analyzed during this study are included in this published article and its supplementary information files. Sanger sequence data to GenBank code BankIt2630585: OP611209—OP611320. Amplicon-based NGS data to ENA, project accession: PRJEB56730 and submission accession: ERA18523751.

## Abbreviations

ANLA: Autoridad Nacional de Licencias Ambientales (National Authority for Environmental Licenses)
INS: Instituto Nacional de Salud (National Health Institute)
NGS: Next-generation sequencing
NTD: Neglected tropical diseases
VBD: Vector-borne diseases
VL: Visceral leishmaniasis
WHO: World Health Organization
