# Supplementary material for: An overview of the trypanosomatid (Kinetoplastida: Trypanosomatidae) parasites infecting several mammal species in Colombia
Source: Parasit Vectors. 2022 Dec 16;15:471. doi: 10.1186/s13071-022-05595-y (PMC9756507; doi:10.1186/s13071-022-05595-y)
Supplement: Supplementary file 1 — Additional file 1: Fig. S1. Map showing the different departments and the capital district of Colombia. [file 13071_2022_5595_MOESM1_ESM.pdf]

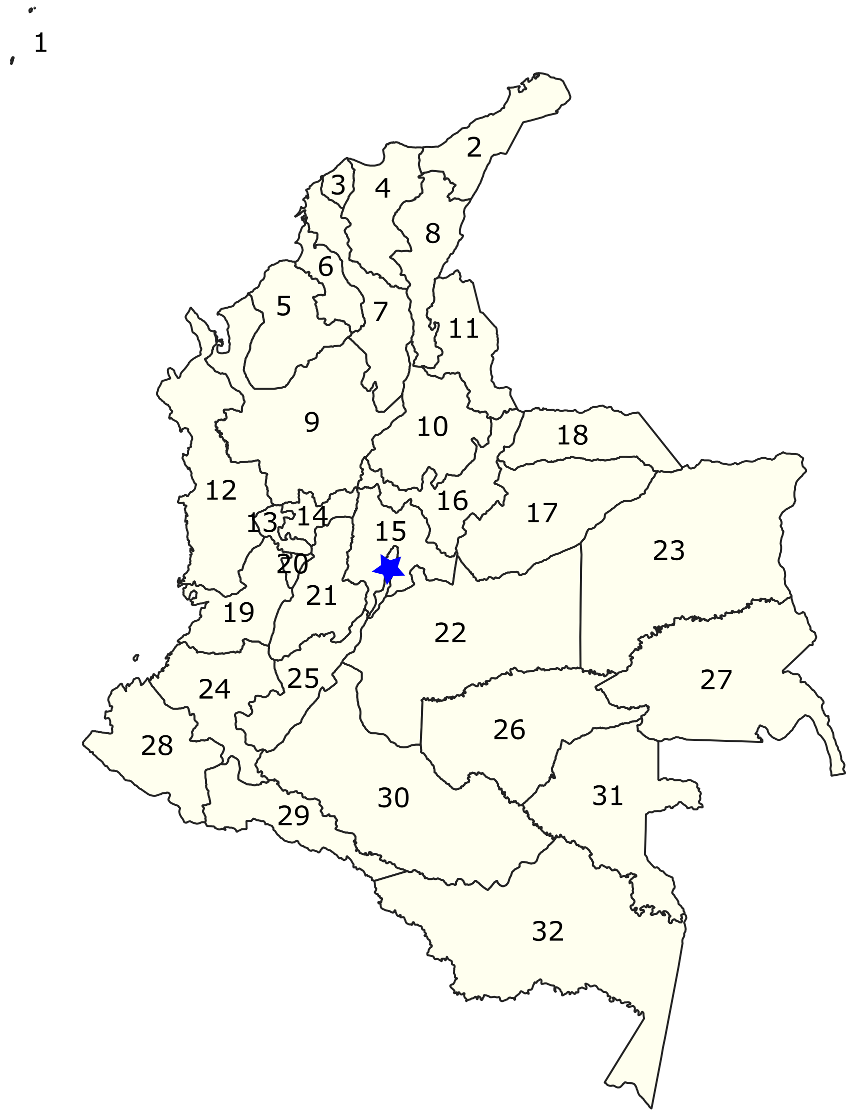

1. Archipielago de San Andrés, Providencia y Santa Catalina
2. La Guajira
3. Atlantico
4. Magdalena
5. Córdoba
6. Sucre
7. Bolívar
8. Cesar
9. Antioquia
10. Santander
11. Norte de Santander
12. Chocó
13. Risaralda
14. Caldas
15. Cundinamarca
16. Boyacá
17. Casanare
18. Arauca
19. Valle del Cauca
20. Quindio
21. Tolima
22. Meta
23. Vichada
24. Cauca
25. Huila
26. Guaviare
27. Guainia
28. Nariño
29. Putumayo
30. Caqueta
31. Vaupés
32. Amazonas

★ Bogotá D. C
