# Supplementary material for: An overview of the trypanosomatid (Kinetoplastida: Trypanosomatidae) parasites infecting several mammal species in Colombia
Source: Parasit Vectors. 2022 Dec 16;15:471. doi: 10.1186/s13071-022-05595-y (PMC9756507; doi:10.1186/s13071-022-05595-y)
Supplement: Supplementary file 2 — Additional file 2: Table S1. Information on collected samples per department, sample code, and mammal species. [file 13071_2022_5595_MOESM2_ESM.pdf]

Table S1. Information of collected samples per department, sample code and mammal especies.

**Origin**	**Sample ID**	***Mammal***
Antioquia	1MCO2	*Canis lupus familiaris*
Antioquia	1A	*Canis lupus familiaris*
Antioquia	1	*Hydrochaeris hydrochaeris*
Antioquia	2	*Hydrochaeris hydrochaeris*
Antioquia	2TC	*Canis lupus familiaris*
Antioquia	3Leish	*Canis lupus familiaris*
Antioquia	3	*Canis lupus familiaris*
Antioquia	3TC	*Canis lupus familiaris*
Antioquia	4	*Proechimys roberti*
Antioquia	4TC	*Canis lupus familiaris*
Antioquia	6	*Hydrochaeris hydrochaeris*
Antioquia	7LV	*Canis lupus familiaris*
Antioquia	7	*Hydrochaeris hydrochaeris*
Antioquia	8LV	*Canis lupus familiaris*
Antioquia	8	*Hydrochaeris hydrochaeris*
Antioquia	9Leish	*Canis lupus familiaris*
Antioquia	9	*Hydrochaeris hydrochaeris*
Antioquia	10	*Hydrochaeris hydrochaeris*
Antioquia	11	*Odocoileus virginianus*
Antioquia	12	*Odocoileus virginianus*
Antioquia	13	*Callicebus cupreus*
Antioquia	14	*Pecari tajacu*
Antioquia	15	*Pecari tajacu*
Antioquia	16	*Pecari tajacu*
Antioquia	17	*Pecari tajacu*
Antioquia	18	*Pecari tajacu*
Antioquia	19	*Pecari tajacu*
Antioquia	20	*Pecari tajacu*
Antioquia	21	*Tapirus terrestris*
Antioquia	22	*Choloepus didactylus*
Antioquia	23	*Choloepus didactylus*
Antioquia	24	*Odocoileus virginianus*
Antioquia	25	*Coendou bicolor*
Antioquia	CO2 Tc	*Chinchilla lanigera*
Santander	MTC1	*Canis lupus familiaris*
Santander	MTC2	*Canis lupus familiaris*
Santander	MTC3	*Canis lupus familiaris*
Santander	MTC4	*Canis lupus familiaris*

Santander	MTC5	*Canis lupus familiaris*
Santander	MTC6	*Canis lupus familiaris*
Santander	MTC7	*Canis lupus familiaris*
Santander	MTC8	*Canis lupus familiaris*
Santander	MTC9	*Canis lupus familiaris*
Santander	MTC10	*Canis lupus familiaris*
Santander	MTC11	*Canis lupus familiaris*
Santander	PC01	*Canis lupus familiaris*
Casanare	PUC02	*Myotis martiniquensis*
Casanare	PUC03	*Desmodus rotundus*
Casanare	PUC07	*Desmodus rotundus*
Casanare	MT27	*Carollia perspicillata*
Casanare	MT37	*Myotis brandtii*
Casanare	MT38	*Myotis brandtii*
Casanare	MT39	*Myotis brandtii*
Casanare	MT43	*Phyllostomus hastatus*
Casanare	MT44	*Phyllostomus hastatus*
Casanare	MT45	*Myotis brandtii*
Casanare	MT46	*Phyllostomus hastatus*
Casanare	MT49	*Phyllostomus hastatus*
Casanare	MT50	*Phyllostomus hastatus*
Casanare	MT52	*Phyllostomus hastatus*
Casanare	MT53	*Phyllostomus hastatus*
Casanare	MT54	*Phyllostomus hastatus*
Casanare	MT56	*Phyllostomus hastatus*
Casanare	MT57	*Phyllostomus hastatus*
Casanare	MT61	*Phyllostomus hastatus*
Casanare	MT62	*Glossophaga soricina*
Casanare	MT63	*Phyllostomus hastatus*
Casanare	MT64	*Phyllostomus hastatus*
Casanare	MT66	*Carollia perspicillata*
Casanare	MT74	*Myotis brandtii*
Casanare	MT75	*Myotis brandtii*
Casanare	MT81	*Phyllostomus elongatus*
Casanare	MT83	*Phyllostomus hastatus*
Casanare	MT87	*Phyllostomus elongatus*
Casanare	MT103	*Glossophaga soricina*
Casanare	MT126	*Carollia brevicauda*
Casanare	MT133	*Glossophaga soricina*
Casanare	MT137	*Carollia brevicauda*
Casanare	MT142	*Carollia brevicauda*
Casanare	MT147	*Carollia brevicauda*
Casanare	MT151	*Carollia brevicauda*

Casanare	MT154	*Carollia brevicauda*
Casanare	MT80	*Phyllostomus hastatus*
Casanare	MT111	*Carollia perspicillata*
Casanare	MT120	*Carollia perspicillata*
Casanare	BT1	*Phyllostomus hastatus*
Casanare	BT2	*Phyllostomus hastatus*
Casanare	BT2a	*Phyllostomus hastatus*
Casanare	BT3	*Phyllostomus hastatus*
Casanare	BT4	*Carollia perspicillata*
Casanare	BTC1	*Glossophaga soricina*
Casanare	BTC2	*Phyllostomus hastatus*
Córdoba	375	*Canis lupus familiaris*
Córdoba	376	*Canis lupus familiaris*
Córdoba	378	*Canis lupus familiaris*
Córdoba	382	*Canis lupus familiaris*
Córdoba	386	*Canis lupus familiaris*
Córdoba	389	*Canis lupus familiaris*
Sucre	390	*Canis lupus familiaris*
Sucre	392	*Canis lupus familiaris*
Sucre	393	*Canis lupus familiaris*
Sucre	395	*Canis lupus familiaris*
Sucre	397	*Canis lupus familiaris*
Sucre	398	*Canis lupus familiaris*
Sucre	399	*Canis lupus familiaris*
Sucre	401	*Canis lupus familiaris*
Sucre	402	*Canis lupus familiaris*
Sucre	403	*Canis lupus familiaris*
La Guajira	718-002	*Canis lupus familiaris*
La Guajira	718-066	*Canis lupus familiaris*
La Guajira	718-488	*Canis lupus familiaris*
La Guajira	719-128	*Canis lupus familiaris*
La Guajira	719-129	*Canis lupus familiaris*
La Guajira	719-130	*Canis lupus familiaris*
La Guajira	719-132	*Canis lupus familiaris*
Casanare	Z2	*Didelphis marsupialis*
Casanare	Z3	*Didelphis marsupialis*
Casanare	Z5	*Didelphis marsupialis*
Huila	C123	*Canis lupus familiaris*
Huila	C120	*Canis lupus familiaris*
Huila	C122	*Canis lupus familiaris*
Huila	C117	*Canis lupus familiaris*
Huila	C111	*Canis lupus familiaris*
Huila	C109	*Canis lupus familiaris*

Huila	C84	*Canis lupus familiaris*
Huila	C77	*Canis lupus familiaris*
Huila	C66	*Canis lupus familiaris*
Huila	C67	*Canis lupus familiaris*
Huila	C718	*Canis lupus familiaris*
Huila	C19	*Canis lupus familiaris*
Huila	C16	*Canis lupus familiaris*
Huila	C09	*Canis lupus familiaris*
Norte de Santander	C03	*Canis lupus familiaris*
Norte de Santander	C04	*Canis lupus familiaris*
Norte de Santander	C46	*Canis lupus familiaris*
Norte de Santander	C40	*Canis lupus familiaris*
Norte de Santander	C29	*Canis lupus familiaris*
Huila	C333	*Canis lupus familiaris*
Huila	C334	*Canis lupus familiaris*
Huila	C335	*Canis lupus familiaris*
Huila	C336	*Canis lupus familiaris*
Huila	C337	*Canis lupus familiaris*
Huila	C338	*Canis lupus familiaris*
Huila	C339	*Canis lupus familiaris*
Huila	C342	*Canis lupus familiaris*
Huila	C343	*Canis lupus familiaris*
Tolima	C371	*Canis lupus familiaris*
Tolima	C372	*Canis lupus familiaris*
Tolima	C373	*Canis lupus familiaris*
Tolima	C374	*Canis lupus familiaris*
Tolima	C375	*Canis lupus familiaris*
Tolima	C376	*Canis lupus familiaris*
Tolima	C377	*Canis lupus familiaris*
Tolima	C378	*Canis lupus familiaris*
Tolima	C379	*Canis lupus familiaris*
César	C403	*Canis lupus familiaris*
La Guajira	717-402	*Homo sapiens sapiens*
Sucre	713-115	*Homo sapiens sapiens*
Huila	712-189	*Homo sapiens sapiens*
Tolima	719-114	*Homo sapiens sapiens*
Santander	718-417	*Homo sapiens sapiens*
Huila	718-736	*Homo sapiens sapiens*
La Guajira	718-402	*Homo sapiens sapiens*
Tolima	719-219	*Homo sapiens sapiens*
Santander	719-193	*Homo sapiens sapiens*
Norte de Santander	720_01	*Homo sapiens sapiens*
Huila	H15	*Homo sapiens sapiens*

Huila	H11	*Homo sapiens sapiens*
Bolívar	H89	*Homo sapiens sapiens*
Bolívar	H57	*Homo sapiens sapiens*
Sucre	H59	*Homo sapiens sapiens*
Huila	H54	*Homo sapiens sapiens*
Córdoba	H02	*Homo sapiens sapiens*
Bolívar	H01	*Homo sapiens sapiens*
