# Supplementary material for: An overview of the trypanosomatid (Kinetoplastida: Trypanosomatidae) parasites infecting several mammal species in Colombia
Source: Parasit Vectors. 2022 Dec 16;15:471. doi: 10.1186/s13071-022-05595-y (PMC9756507; doi:10.1186/s13071-022-05595-y)
Supplement: Supplementary file 3 — Additional file 3: Table S2. BLASTn results for the 337bp Hsp70 gene fragment of the samples analyzed herein. [file 13071_2022_5595_MOESM3_ESM.pdf]

Table S2. Blastn results for the 337pb *Hsp70* gene fragment of the samples herein analyzed

Sample ID	Country	Department	Municipality	Coordinates_Municipality	Mammal	*Hsp70*_Leish
1MCO2	Colombia	Antioquia	Urrao	6.416455414982666, -76.34382862356753	*Canis_lupus_familiaris*	*Leishmania_spp*
1A	Colombia	Antioquia	Urrao	6.416455414982666, -76.34382862356753	*Canis_lupus_familiaris*	*Leishmania_spp*
1	Colombia	Antioquia	Urrao	6.416455414982666, -76.34382862356753	*Hydrochaeris_hydrochaeris*	*Leishmania_panamensis*
2	Colombia	Antioquia	Urrao	6.416455414982666, -76.34382862356753	*Hydrochaeris_hydrochaeris*	*Leishmania_amazonensis*
2TC	Colombia	Antioquia	Urrao	6.416455414982666, -76.34382862356753	*Canis_lupus_familiaris*	*Leishmania_amazonensis*
3Leish	Colombia	Antioquia	Urrao	6.416455414982666, -76.34382862356753	*Canis_lupus_familiaris*	*Trypanosoma_cruzi*
3	Colombia	Antioquia	Urrao	6.416455414982666, -76.34382862356753	*Canis_lupus_familiaris*	*Leishmania_spp*
3TC	Colombia	Antioquia	Urrao	6.416455414982666, -76.34382862356753	*Canis_lupus_familiaris*	*Leishmania_panamensis*
4	Colombia	Antioquia	Urrao	6.416455414982666, -76.34382862356753	*Proechimys_roberti*	*Leishmania_spp*
4TC	Colombia	Antioquia	Urrao	6.416455414982666, -76.34382862356753	*Canis_lupus_familiaris*	*Leishmania_amazonensis*
6	Colombia	Antioquia	Urrao	6.416455414982666, -76.34382862356753	*Hydrochaeris_hydrochaeris*	*Leishmania_spp*
7LV	Colombia	Antioquia	Urrao	6.416455414982666, -76.34382862356753	*Canis_lupus_familiaris*	*Leishmania_spp*
7	Colombia	Antioquia	Urrao	6.416455414982666, -76.34382862356753	*Hydrochaeris_hydrochaeris*	*Leishmania_spp*
8LV	Colombia	Antioquia	Urrao	6.416455414982666, -76.34382862356753	*Canis_lupus_familiaris*	*Leishmania_amazonensis*
8	Colombia	Antioquia	Urrao	6.416455414982666, -76.34382862356753	*Hydrochaeris_hydrochaeris*	*Leishmania_spp*
9Leish	Colombia	Antioquia	Urrao	6.416455414982666, -76.34382862356753	*Canis_lupus_familiaris*	*Trypanosoma_cruzi*
9	Colombia	Antioquia	Urrao	6.416455414982666, -76.34382862356753	*Hydrochaeris_hydrochaeris*	*Leishmania_spp*
10	Colombia	Antioquia	Urrao	6.416455414982666, -76.34382862356753	*Hydrochaeris_hydrochaeris*	*Leishmania_amazonensis*
11	Colombia	Antioquia	Urrao	6.416455414982666, -76.34382862356753	*Odocoileus_virginianus*	*Leishmania_amazonensis*
12	Colombia	Antioquia	Urrao	6.416455414982666, -76.34382862356753	*Odocoileus_virginianus*	*Leishmania_amazonensis*
13	Colombia	Antioquia	Urrao	6.416455414982666, -76.34382862356753	*Callicebus_cupreus*	*Leishmania_spp*
14	Colombia	Antioquia	Urrao	6.416455414982666, -76.34382862356753	*Pecari_tajacu*	*Leishmania_panamensis*
15	Colombia	Antioquia	Urrao	6.416455414982666, -76.34382862356753	*Pecari_tajacu*	*Leishmania_spp*
16	Colombia	Antioquia	Urrao	6.416455414982666, -76.34382862356753	*Pecari_tajacu*	*Leishmania_amazonensis*
17	Colombia	Antioquia	Urrao	6.416455414982666, -76.34382862356753	*Pecari_tajacu*	*Leishmania_spp*
18	Colombia	Antioquia	Urrao	6.416455414982666, -76.34382862356753	*Pecari_tajacu*	*Leishmania_spp*
19	Colombia	Antioquia	Urrao	6.416455414982666, -76.34382862356753	*Pecari_tajacu*	*Leishmania_infantum*
20	Colombia	Antioquia	Urrao	6.416455414982666, -76.34382862356753	*Pecari_tajacu*	*Leishmania_panamensis*
21	Colombia	Antioquia	Urrao	6.416455414982666, -76.34382862356753	*Tapirus_terrestris*	*Leishmania_spp*
22	Colombia	Antioquia	Urrao	6.416455414982666, -76.34382862356753	*Choloepus_didactylus*	*Leishmania_infantum*

23	Colombia	Antioquia	Urrao	6.416455414982666, -76.34382862356753	*Choloepus_didactylus*	*Leishmania_spp*
24	Colombia	Antioquia	Urrao	6.416455414982666, -76.34382862356753	*Odocoileus_virginianus*	*Leishmania_spp*
CO2_Tc	Colombia	Antioquia	Urrao	6.416455414982666, -76.34382862356753	*Chinchilla_lanigera*	*Trypanosoma_cruzi*
H57	Colombia	Bolívar	El Carmen de Bolívar	9.70681100758877, -75.11436013376944	*Homo_sapiens_sapiens*	*Leishmania_amazonensis*
H89	Colombia	Bolívar	El Carmen de Bolívar	9.70681100758877, -75.11436013376944	*Homo_sapiens_sapiens*	*Leishmania_infantum*
PC01	Colombia	Santander	PiedeCuesta	6.990807134758075, -73.05330127555473	*Canis_lupus_familiaris*	*Leishmania_infantum*
PUC02	Colombia	Casanare	Maní	4.819354162123441, -72.28181813471689	*Myotis_martiniquensis*	*Trypanosoma_cruzi*
PUC03	Colombia	Casanare	Maní	4.819354162123441, -72.28181813471689	*Desmodus_rotundus*	*Trypanosoma_cruzi*
PUC07	Colombia	Casanare	Maní	4.819354162123441, -72.28181813471689	*Desmodus_rotundus*	*Trypanosoma_cruzi*
MT37	Colombia	Casanare	Maní	4.819354162123441, -72.28181813471689	*Myotis_brandtii*	*Trypanosoma_cruzi*
MT38	Colombia	Casanare	Maní	4.819354162123441, -72.28181813471689	*Myotis_brandtii*	*Trypanosoma_cruzi*
MT39	Colombia	Casanare	Maní	4.819354162123441, -72.28181813471689	*Myotis_brandtii*	*Trypanosoma_cruzi*
MT43	Colombia	Casanare	Maní	4.819354162123441, -72.28181813471689	*Phyllostomus_hastatus*	*Trypanosoma_cruzi*
MT45	Colombia	Casanare	Maní	4.819354162123441, -72.28181813471689	*Myotis_brandtii*	*Trypanosoma_cruzi*
MT49	Colombia	Casanare	Maní	4.819354162123441, -72.28181813471689	*Phyllostomus_hastatus*	*Trypanosoma_cruzi*
MT50	Colombia	Casanare	Maní	4.819354162123441, -72.28181813471689	*Phyllostomus_hastatus*	*Trypanosoma_cruzi*
MT52	Colombia	Casanare	Maní	4.819354162123441, -72.28181813471689	*Phyllostomus_hastatus*	*Trypanosoma_cruzi*
MT53	Colombia	Casanare	Maní	4.819354162123441, -72.28181813471689	*Phyllostomus_hastatus*	*Trypanosoma_cruzi*
MT56	Colombia	Casanare	Maní	4.819354162123441, -72.28181813471689	*Phyllostomus_hastatus*	*Trypanosoma_cruzi*
MT57	Colombia	Casanare	Maní	4.819354162123441, -72.28181813471689	*Phyllostomus_hastatus*	*Trypanosoma_cruzi*
MT61	Colombia	Casanare	Maní	4.819354162123441, -72.28181813471689	*Phyllostomus_hastatus*	*Trypanosoma_cruzi*
MT63	Colombia	Casanare	Maní	4.819354162123441, -72.28181813471689	*Phyllostomus_hastatus*	*Trypanosoma_cruzi*
MT64	Colombia	Casanare	Maní	4.819354162123441, -72.28181813471689	*Phyllostomus_hastatus*	*Trypanosoma_cruzi*
MT66	Colombia	Casanare	Maní	4.819354162123441, -72.28181813471689	*Carollia_perspicillata*	*Trypanosoma_cruzi*
MT74	Colombia	Casanare	Maní	4.819354162123441, -72.28181813471689	*Myotis_brandtii*	*Trypanosoma_cruzi*
MT83	Colombia	Casanare	Maní	4.819354162123441, -72.28181813471689	*Phyllostomus_hastatus*	*Trypanosoma_cruzi*
MT103	Colombia	Casanare	Maní	4.819354162123441, -72.28181813471689	*Glossophaga_soricina*	*Trypanosoma_cruzi*
MT142	Colombia	Casanare	Maní	4.819354162123441, -72.28181813471689	*Glossophaga_soricina*	*Trypanosoma_cruzi*
BT1	Colombia	Casanare	Maní	4.819354162123441, -72.28181813471689	*Phyllostomus_hastatus*	*Leishmania_braziliensis*
BT2	Colombia	Casanare	Maní	4.819354162123441, -72.28181813471689	*Phyllostomus_hastatus*	*Leishmania_spp*
BT2a	Colombia	Casanare	Maní	4.819354162123441, -72.28181813471689	*Phyllostomus_hastatus*	*Leishmania_spp*
BT3	Colombia	Casanare	Maní	4.819354162123441, -72.28181813471689	*Phyllostomus_hastatus*	*Leishmania_braziliensis*
BTC1	Colombia	Casanare	Maní	4.819354162123441, -72.28181813471689	*Glossophaga_soricina*	*Leishmania_amazonensis*
BTC2	Colombia	Casanare	Maní	4.819354162123441, -72.28181813471689	*Phyllostomus_hastatus*	*Leishmania_braziliensis*

Z3	Colombia	Casanare	Maní	4.819354162123441, -72.28181813471689	*Didelphis_marsupialis*	*Trypanosoma_cruzi*
Z5	Colombia	Casanare	Maní	4.819354162123441, -72.28181813471689	*Didelphis_marsupialis*	*Trypanosoma_cruzi*
H02	Colombia	Córdoba	San Andrés de Sotavento	9.145724514435896, -75.5083324239828	*Homo_sapiens_sapiens*	*Leishmania_infantum*
375	Colombia	Córdoba	Tuchín	9.193640822683856, -75.54077383320312	*Canis_lupus_familiaris*	*Trypanosoma_cruzi*
376	Colombia	Córdoba	Tuchín	9.193640822683856, -75.54077383320312	*Canis_lupus_familiaris*	*Leishmania_amazonensis*
378	Colombia	Córdoba	Tuchín	9.193640822683856, -75.54077383320312	*Canis_lupus_familiaris*	*Leishmania_braziliensis*
382	Colombia	Córdoba	Tuchín	9.193640822683856, -75.54077383320312	*Canis_lupus_familiaris*	*Leishmania_braziliensis*
386	Colombia	Córdoba	Tuchín	9.193640822683856, -75.54077383320312	*Canis_lupus_familiaris*	*Leishmania_braziliensis*
389	Colombia	Córdoba	Tuchín	9.193640822683856, -75.54077383320312	*Canis_lupus_familiaris*	*Leishmania_braziliensis*
718_402	Colombia	La_Guajira	Hatonuevo	11.067711783701261, -72.76042612314019	*Homo_sapiens_sapiens*	*Leishmania_spp*
712_189	Colombia	Huila	Huila	2.936245710824883, -75.26930319642237	*Homo_sapiens_sapiens*	*Leishmania_infantum*
718_736	Colombia	Huila	Huila	2.936245710824883, -75.26930319642237	*Homo_sapiens_sapiens*	*Leishmania_infantum*
C334	Colombia	Huila	Huila	2.936245710824883, -75.26930319642237	*Canis_lupus_familiaris*	*Leishmania_infantum*
C335	Colombia	Huila	Huila	2.936245710824883, -75.26930319642237	*Canis_lupus_familiaris*	*Leishmania_infantum*
718_002	Colombia	La_Guajira	Hatonuevo	11.067711783701261, -72.76042612314019	*Canis_lupus_familiaris*	*Leishmania_panamensis*
718_066	Colombia	La_Guajira	Hatonuevo	11.067711783701261, -72.76042612314019	*Canis_lupus_familiaris*	*Leishmania_amazonensis*
718_488	Colombia	La_Guajira	Hatonuevo	11.067711783701261, -72.76042612314019	*Canis_lupus_familiaris*	*Leishmania_spp*
719_128	Colombia	La_Guajira	Hatonuevo	11.067711783701261, -72.76042612314019	*Canis_lupus_familiaris*	*Leishmania_amazonensis*
719_129	Colombia	La_Guajira	Hatonuevo	11.067711783701261, -72.76042612314019	*Canis_lupus_familiaris*	*Leishmania_amazonensis*
719_130	Colombia	La_Guajira	Hatonuevo	11.067711783701261, -72.76042612314019	*Canis_lupus_familiaris*	*Leishmania_amazonensis*
719_132	Colombia	La_Guajira	Hatonuevo	11.067711783701261, -72.76042612314019	*Canis_lupus_familiaris*	*Leishmania_amazonensis*
717_402	Colombia	La_Guajira	Hatonuevo	11.067711783701261, -72.76042612314019	*Homo_sapiens_sapiens*	*Leishmania_amazonensis*
720_01	Colombia	Norte_Santander			*Homo_sapiens_sapiens*	*Leishmania_infantum*
MTC1	Colombia	Santander	Piedecuesta	6.990807134758075, -73.05330127555473	*Canis_lupus_familiaris*	*Leishmania_spp*
MTC2	Colombia	Santander	Piedecuesta	6.990807134758075, -73.05330127555473	*Canis_lupus_familiaris*	*Trypanosoma_cruzi*
MTC3	Colombia	Santander	Piedecuesta	6.990807134758075, -73.05330127555473	*Canis_lupus_familiaris*	*Leishmania_spp*
MTC4	Colombia	Santander	Piedecuesta	6.990807134758075, -73.05330127555473	*Canis_lupus_familiaris*	*Leishmania_spp*
MTC5	Colombia	Santander	Piedecuesta	6.990807134758075, -73.05330127555473	*Canis_lupus_familiaris*	*Leishmania_spp*
MTC6	Colombia	Santander	Piedecuesta	6.990807134758075, -73.05330127555473	*Canis_lupus_familiaris*	*Leishmania_amazonensis*
MTC7	Colombia	Santander	Piedecuesta	6.990807134758075, -73.05330127555473	*Canis_lupus_familiaris*	*Trypanosoma_cruzi*
MTC8	Colombia	Santander	Piedecuesta	6.990807134758075, -73.05330127555473	*Canis_lupus_familiaris*	*Leishmania_amazonensis*
MTC9	Colombia	Santander	Piedecuesta	6.990807134758075, -73.05330127555473	*Canis_lupus_familiaris*	*Leishmania_spp*
MTC10	Colombia	Santander	Piedecuesta	6.990807134758075, -73.05330127555473	*Canis_lupus_familiaris*	*Leishmania_panamensis*
718_417	Colombia	Santander			*Homo_sapiens_sapiens*	*Leishmania_infantum*
719_193	Colombia	Santander	Girón	7.075002072113353, -73.16920373823982	*Homo_sapiens_sapiens*	*Leishmania_amazonensis*

390	Colombia	Sucre	Sincelejo	9.309807706650341, -75.40101029361902	*Canis_lupus_familiaris*	*Leishmania_braziliensis*
392	Colombia	Sucre	Sincelejo	9.309807706650341, -75.40101029361902	*Canis_lupus_familiaris*	*Leishmania_braziliensis*
393	Colombia	Sucre	Sincelejo	9.309807706650341, -75.40101029361902	*Canis_lupus_familiaris*	*Leishmania_amazonensis*
395	Colombia	Sucre	Sincelejo	9.309807706650341, -75.40101029361902	*Canis_lupus_familiaris*	*Trypanosoma_cruzi*
397	Colombia	Sucre	Sincelejo	9.309807706650341, -75.40101029361902	*Canis_lupus_familiaris*	*Leishmania_braziliensis*
398	Colombia	Sucre	Sincelejo	9.309807706650341, -75.40101029361902	*Canis_lupus_familiaris*	*Leishmania_braziliensis*
399	Colombia	Sucre	Sincelejo	9.309807706650341, -75.40101029361902	*Canis_lupus_familiaris*	*Leishmania_panamensis*
401	Colombia	Sucre	Sincelejo	9.309807706650341, -75.40101029361902	*Canis_lupus_familiaris*	*Leishmania_panamensis*
403	Colombia	Sucre	Sincelejo	9.309807706650341, -75.40101029361902	*Canis_lupus_familiaris*	*Leishmania_panamensis*
713_115	Colombia	Sucre	Ovejas	9.523907484665221, -75.22959085388135	*Homo_sapiens_sapiens*	*Leishmania_infantum*
H59	Colombia	Sucre	Sincelejo	9.309807706650341, -75.40101029361902	*Homo_sapiens_sapiens*	*Leishmania_infantum*
719_114	Colombia	Tolima			*Homo_sapiens_sapiens*	*Leishmania_infantum*
719_100	Colombia	Tolima	Chaparral	3.7224458298518512, -75.47611545321647	*Homo_sapiens_sapiens*	*Leishmania_infantum*
