# Supplementary material for: An overview of the trypanosomatid (Kinetoplastida: Trypanosomatidae) parasites infecting several mammal species in Colombia
Source: Parasit Vectors. 2022 Dec 16;15:471. doi: 10.1186/s13071-022-05595-y (PMC9756507; doi:10.1186/s13071-022-05595-y)
Supplement: Supplementary file 4 — Additional file 4: Table S3. Shannon and Simpson index values from the different species found in the analyzed samples by amplicon-based NGS. [file 13071_2022_5595_MOESM4_ESM.pdf]

Additional file 4: Table S3. Shannon and Simpson indices values from the different species found in the analyzed samples by amplicon-based NGS.

X	*T. cruzi*	*T. rangeli*	*L. infantum*	*L. amazonensis*	*L. mexicana*	*L. braziliensis*	*L. panamensis*	*L. naiffi*	*L. lindenbergi*	SHANNON	SIMPSON	INVSIMP	Pielou's evenness
H02	0	0	100	0	0	0	0	0	0	0	0	1	NA
H59	87	0	11	0	0	0	0	0	0	0.3511833	0.199292	1.248895	0.5066503
H78	0	0	0	10	0	33	50	4	3	1.1766425	0.6286	2.692515	0.7310891
H79	0	0	100	0	0	0	0	0	0	0	0	1	NA
H83	0	0	16	0	0	33	47	4	0	1.1426873	0.643	2.80112	0.8242747
H85	0	0	65	35	0	0	0	0	0	0.6474466	0.455	1.834862	0.9340681
H86	0	0	80	20	0	0	0	0	0	0.5004024	0.32	1.470588	0.7219281
MT43	98	0	0	0	0	0	1	0	0	0.0564652	0.019998	1.020406	0.081462
MT53	99	0	0	0	0	0	0	0	0	0	0	1	NA
MT56	98	0	0	0	0	0	0	0	0	0	0	1	NA
MT59	94	0	0	0	0	3	2	0	0	0.2339901	0.097133	1.107583	0.212987
MT63	100	0	0	0	0	0	0	0	0	0	0	1	NA
MT83	98	0	0	0	0	1	0	0	0	0.0564652	0.019998	1.020406	0.081462
PC01	0	0	100	0	0	0	0	0	0	0	0	1	NA
PUC02	100	0	0	0	0	0	0	0	0	0	0	1	NA
PUC03	100	0	0	0	0	0	0	0	0	0	0	1	NA
PUC07	80	0	0	0	0	20	0	0	0	0.5004024	0.32	1.470588	0.7219281
MTC2	100	0	0	0	0	0	0	0	0	0	0	1	NA
MTC7	20	56	24	0	0	0	0	0	0	0.9890939	0.5888	2.431907	0.900312
R333	0	0	100	0	0	0	0	0	0	0	0	1	NA
R94	0	0	0	0	0	31	48	21	0	1.043108	0.6294	2.698327	0.9494778
R95	0	0	0	0	0	2	88	10	0	0.4209923	0.2152	1.27421	0.3832037
SAC382	98	0	0	0	0	0	0	0	0	0	0	1	NA
SAC398	39	0	0	12	0	48	0	0	0	0.9737518	0.595041	2.469388	0.8863471
SAC401	62	0	5	0	0	33	0	0	0	0.8120275	0.5042	2.016942	0.7391393
SC334	94	0	0	0	0	0	0	0	0	0	0	1	NA
SC335	0	0	0	0	99	0	0	0	0	0	0	1	NA
